# Development of a Multilevel Model to Identify Patients at Risk for Delay in Starting Cancer Treatment

**DOI:** 10.1001/jamanetworkopen.2023.28712

**Published:** 2023-08-14

**Authors:** Zachary A. K. Frosch, Jill Hasler, Elizabeth Handorf, Tesla DuBois, Richard J. Bleicher, Martin J. Edelman, Daniel M. Geynisman, Michael J. Hall, Carolyn Y. Fang, Shannon M. Lynch

**Affiliations:** 1 Department of Hematology/Oncology, Fox Chase Cancer Center, Philadelphia, Pennsylvania; 2 Cancer Prevention and Control Research Program, Fox Chase Cancer Center, Philadelphia, Pennsylvania; 3 Department of Biostatistics, Fox Chase Cancer Center, Philadelphia, Pennsylvania; 4 Department of Surgical Oncology, Fox Chase Cancer Center, Philadelphia, Pennsylvania

## Abstract

**Importance:**

Delays in starting cancer treatment disproportionately affect vulnerable populations and can influence patients’ experience and outcomes. Machine learning algorithms incorporating electronic health record (EHR) data and neighborhood-level social determinants of health (SDOH) measures may identify at-risk patients.

**Objective:**

To develop and validate a machine learning model for estimating the probability of a treatment delay using multilevel data sources.

**Design, Setting, and Participants:**

This cohort study evaluated 4 different machine learning approaches for estimating the likelihood of a treatment delay greater than 60 days (group least absolute shrinkage and selection operator [LASSO], bayesian additive regression tree, gradient boosting, and random forest). Criteria for selecting between approaches were discrimination, calibration, and interpretability/simplicity. The multilevel data set included clinical, demographic, and neighborhood-level census data derived from the EHR, cancer registry, and American Community Survey. Patients with invasive breast, lung, colorectal, bladder, or kidney cancer diagnosed from 2013 to 2019 and treated at a comprehensive cancer center were included. Data analysis was performed from January 2022 to June 2023.

**Exposures:**

Variables included demographics, cancer characteristics, comorbidities, laboratory values, imaging orders, and neighborhood variables.

**Main Outcomes and Measures:**

The outcome estimated by machine learning models was likelihood of a delay greater than 60 days between cancer diagnosis and treatment initiation. The primary metric used to evaluate model performance was area under the receiver operating characteristic curve (AUC-ROC).

**Results:**

A total of 6409 patients were included (mean [SD] age, 62.8 [12.5] years; 4321 [67.4%] female; 2576 [40.2%] with breast cancer, 1738 [27.1%] with lung cancer, and 1059 [16.5%] with kidney cancer). A total of 1621 (25.3%) experienced a delay greater than 60 days. The selected group LASSO model had an AUC-ROC of 0.713 (95% CI, 0.679-0.745). Lower likelihood of delay was seen with diagnosis at the treating institution; first malignant neoplasm; Asian or Pacific Islander or White race; private insurance; and lacking comorbidities. Greater likelihood of delay was seen at the extremes of neighborhood deprivation. Model performance (AUC-ROC) was lower in Black patients, patients with race and ethnicity other than non-Hispanic White, and those living in the most disadvantaged neighborhoods. Though the model selected neighborhood SDOH variables as contributing variables, performance was similar when fit with and without these variables.

**Conclusions and Relevance:**

In this cohort study, a machine learning model incorporating EHR and SDOH data was able to estimate the likelihood of delays in starting cancer therapy. Future work should focus on additional ways to incorporate SDOH data to improve model performance, particularly in vulnerable populations.

## Key Points

**Question:**

Can machine learning algorithms incorporating electronic health record data and neighborhood-level social determinants of health measures identify patients at risk of a delay in starting cancer treatment?

**Findings:**

In this cohort study including 6409 patients to evaluate 4 different machine learning approaches for estimating the risk of a delay greater than 60 days between a cancer diagnosis and the start of treatment, a group least absolute shrinkage and selection operator model was able to estimate the likelihood of a treatment delay with an area under the receiver operating characteristic curve of 0.713.

**Meaning:**

Machine learning models incorporating multilevel data sources can estimate the likelihood of delays in starting cancer therapy.

## Teaser

This cohort study develops and validates a machine learning model for estimating the probability of a treatment delay among patients starting treatment for cancer.

## Introduction

Delays in the initiation of patients’ cancer treatment can worsen clinical outcomes,^1,2,3,4^ cause distress,^5,6^ and exacerbate disparities in care.^1,7,8^ Yet patients have been experiencing longer delays with greater frequency.^1,9,10^ Delays more often affect vulnerable populations, including patients from underserved racial and ethnic groups,^1,8^ those with lower incomes,^7,11^ and those living in low-resource, highly deprived neighborhoods. Thus, the factors contributing to delays are nuanced, multilevel, and often influenced not only by clinical factors, but social determinants of health (SDOH). Reflecting the importance of timely cancer treatment and its contribution to health disparities, certain accrediting organizations now consider it a quality metric,^12,13,14^ and investigators have also used it as an outcome to evaluate policy interventions.^9,15^

Accurately determining which patients are at risk for treatment delays may facilitate timely and effective interventions. While care coordination and patient navigation services may improve the timeliness of cancer treatment,^7,16,17^ these interventions are resource intensive.^16^ Therefore, identifying patients at the greatest risk of delay at diagnosis and subsequently targeting them for interventions could provide the greatest return on this investment and have the added benefit of identifying patients who also are affected by health disparities. Yet most studies of treatment delays to date have focused on association with survival^1,2^; have not simultaneously considered clinical, demographic, and SDOH indicators; or have approached the issue within an explanatory framework^8,11,18^ rather than a predictive one.^19^

Predictive modeling approaches, including machine learning algorithms trained using data derived from the electronic health record (EHR), can successfully identify clinically relevant outcomes in patients with cancer.^20,21^ However, it remains unknown whether machine learning algorithms incorporating EHR clinical and demographic data alone or in combination with neighborhood-level SDOH measures derived from publicly available sources can distinguish, or together improve, the identification of patients at risk of delay in starting cancer treatment. Therefore, we aimed to develop and validate a model that estimates the probability of a treatment delay in patients with common solid tumors using multilevel data sources.

## Methods

### Population and Data Sources

This study was approved by the Fox Chase Cancer Center Institutional Review Board (#21-9066), which also determined that it met criteria for a waiver of informed consent. Adult patients diagnosed with invasive breast, lung, colorectal, bladder, or kidney cancer between January 1, 2013, and December 31, 2019, and subsequently treated at our center were eligible. Though prostate cancer is common, patients with prostate cancer were excluded given that this can frequently be managed with an active observation strategy. Similarly, so as not to include those who may have been selected for active observation for an included cancer, patients not treated within 1 year of diagnosis were also excluded. A total of 9918 recorded diagnoses were identified using the institutional cancer registry. A patient flow diagram is presented in eFigure 1 in Supplement 1. Patients were eligible if the index diagnosis was not their first malignant neoplasm; however, each patient was only included in the data set once. Therefore, for some individuals who had multiple qualifying cancers treated at our center, we excluded subsequent qualifying diagnoses. The study data set was derived by combining data from the cancer registry (eg, diagnosis to first treatment interval), the EHR (eg, laboratory values and comorbidity diagnoses), and neighborhood level variables derived from the American Community Survey 5-year estimates based on data collected from 2015 to 2019. To obtain neighborhood-level variables, participant addresses were geocoded to the census tract level using ArcGIS (Esri). Neighborhood-level variables linked to these geocodes (eg, median household income) were then appended to the data set. This resulted in a merged, multilevel data set containing clinical, demographic, and neighborhood-level census data.

### Model Variables

As shown in Table 1, variables were included in several different categories: demographics (including race and ethnicity, which were derived from the EHR), cancer characteristics (eg, tumor type and stage), comorbidities, laboratory values, imaging orders, and neighborhood-level variables. Neighborhood variables were representative of the 5 domains of SDOH that have been found to be associated with cancer outcomes in the literature as described in eMethods in Supplement 1. The full list of included variables along with further details on their derivation and form (eg, continuous vs categorical and, if categorical, the variable groupings) are reported in eMethods in Supplement 1.

**Table 1.** Study Population

Characteristic	Patients, No. (%)		
	No treatment delay (n = 4788)	Treatment delay (n = 1621)	Total (n = 6409)
Primary site			
Breast	2054 (42.9)	522 (32.2)	2576 (40.2)
Colon	509 (10.6)	76 (4.7)	585 (9.1)
Kidney	690 (14.4)	369 (22.8)	1059 (16.5)
Lung	1172 (24.5)	566 (34.9)	1738 (27.1)
Rectum	197 (4.1)	72 (4.4)	269 (4.2)
Bladder	166 (3.5)	16 (1.0)	182 (2.8)
Age, mean (SD), y	62.2 (12.4)	64.7 (12.5)	62.8 (12.5)
Diagnosed at treating institution			
No	3572 (74.6)	1372 (84.6)	4944 (77.1)
Yes	1216 (25.4)	249 (15.4)	1465 (22.9)
First malignant neoplasm			
No	846 (17.7)	418 (25.8)	1264 (19.7)
Yes	3942 (82.3)	1203 (74.2)	5145 (80.3)
Sex			
Female	3290 (68.7)	1031 (63.6)	4321 (67.4)
Male	1498 (31.3)	590 (36.4)	2088 (32.6)
Race^a^			
Asian/Pacific Islander	187 (3.9)	42 (2.6)	229 (3.6)
Black	557 (11.6)	293 (18.1)	850 (13.3)
White	3754 (78.4)	1221 (75.3)	4975 (77.6)
Other	95 (2.0)	29 (1.8)	124 (1.9)
Unknown	195 (4.1)	36 (2.2)	231 (3.6)
Ethnicity^a^			
Hispanic/Latinx	122 (2.5)	44 (2.7)	166 (2.6)
Non-Hispanic/Latinx	4614 (96.4)	1562 (96.4)	6176 (96.4)
Unknown	52 (1.1)	15 (0.9)	67 (1.0)
Primary language			
English	4348 (90.8)	1482 (91.4)	5830 (91.0)
Not English	296 (6.2)	90 (5.6)	386 (6.0)
Unknown	144 (3.0)	49 (3.0)	193 (3.0)
Insurance			
Private	1925 (40.2)	464 (28.6)	2389 (37.3)
Medicaid	270 (5.6)	109 (6.7)	379 (5.9)
Medicare	2320 (48.5)	918 (56.6)	3238 (50.5)
None	159 (3.3)	95 (5.9)	254 (4.0)
Other	114 (2.4)	35 (2.2)	149 (2.3)
Comorbidities			
Congestive heart failure	55 (1.1)	58 (3.6)	113 (1.8)
Cardiac arrhythmia	371 (7.7)	180 (11.1)	551 (8.6)
Valvular heart disease	90 (1.9)	42 (2.6)	132 (2.1)
Pulmonary circulation disorders	62 (1.3)	36 (2.2)	98 (1.5)
Peripheral vascular disorder	89 (1.9)	82 (5.1)	171 (2.7)
Hypertension	1337 (27.9)	694 (42.8)	2031 (31.7)
Paralysis	5 (0.1)	3 (0.2)	8 (0.1)
Neurologic disorder	45 (0.9)	23 (1.4)	68 (1.1)
Chronic pulmonary disease	614 (12.8)	393 (24.2)	1007 (15.7)
Diabetes	412 (8.6)	233 (14.4)	645 (10.1)
Hypothyroidism	230 (4.8)	110 (6.8)	340 (5.3)
Kidney failure	154 (3.2)	120 (7.4)	274 (4.3)
Liver disease	234 (4.9)	97 (6.0)	331 (5.2)
Peptic ulcer disease	9 (0.2)	8 (0.5)	17 (0.3)
HIV/AIDS	6 (0.1)	5 (0.3)	11 (0.2)
Collagen vascular disease	52 (1.1)	29 (1.8)	81 (1.3)
Coagulopathy	24 (0.5)	16 (1.0)	40 (0.6)
Obesity	284 (5.9)	147 (9.1)	431 (6.7)
Weight loss	74 (1.5)	35 (2.2)	109 (1.7)
Fluid and electrolyte disorders	89 (1.9)	44 (2.7)	133 (2.1)
Blood loss anemia	40 (0.8)	16 (1.0)	56 (0.9)
Deficiency anemia	66 (1.4)	27 (1.7)	93 (1.5)
Drug abuse	24 (0.5)	17 (1.0)	41 (0.6)
Psychoses	11 (0.2)	8 (0.5)	19 (0.3)
Depression	127 (2.7)	74 (4.6)	201 (3.1)
Laboratory values, mean (SD)			
Creatinine (mg/dL)	0.88 (0.46)	0.98 (0.76)	0.91 (0.57)
Bilirubin (mg/dL)	0.58 (0.40)	0.57 (0.27)	0.58 (0.37)
Albumin (g/dL)	4.1 (0.47)	4.1 (0.41)	4.1 (0.45)
AST (U/L)	28.4 (35.1)	26.4 (14.7)	27.8 (30.7)
ALT (U/L)	33 (62.8)	29.9 (20.1)	32.1 (54.2)
WBCs (per μL)	8300 (3.4)	7900 (2.9)	8200 (3.3)
Hemoglobin (g/dL)	13 (1.7)	13.1 (1.2)	13. (1.7)
Platelets (10^3^/μL)	282.2 (97.7)	266.6 (93.1)	277.8 (96.7)
Neighborhood variables			
Median household income, mean (SD), USD $	77 863.0 (33 168.3)	76 994.4 (31 151.8)	77 644.5 (32 672.4)
Median household income missing	165 (3.4)	67 (4.1)	232 (3.6)
Percentage below poverty level, mean (SD)	10.7 (10.2)	10.3 (9.5)	10.6 (10)
Percentage below poverty level missing	164 (3.4)	67 (4.1)	231 (3.6)
Percentage renter occupied households, mean (SD)	28.1 (18.4)	27.9 (18.3)	28.1 (18.4)
Percentage renter occupied households missing	165 (3.4)	67 (4.1)	232 (3.6)
Percentage born outside of US, mean (SD)	11.8 (10)	11.3 (9.7)	11.6 (9.9)
Percentage born outside of US missing	164 (3.4)	67 (4.1)	231 (3.6)
Percentage with less than high school education, mean (SD)	9.2 (7.4)	9 (6.7)	9.2 (7.2)
Percent with less than high school education missing	164 (3.4)	67 (4.1)	231 (3.6)
Percentage employed			
Mean (SD)	94.1 (4.1)	94.2 (4.3)	94.1 (4.2)
Missing	164 (3.4)	67 (4.1)	231 (3.6)
Percentage commuting by public transit			
Mean (SD)	8.7 (10.6)	7.9 (9.8)	8.5 (10.4)
Missing	164 (3.4)	67 (4.1)	231 (3.6)
Percentage living in same house as of 1 y ago			
Mean (SD)	89.8 (5.7)	89.7 (5.6)	89.7 (5.6)
Missing	164 (3.4)	67 (4.1)	231 (3.6)
Yost Index quintile			
1	597 (12.5)	197 (12.2)	794 (12.4)
2	638 (13.3)	196 (12.1)	834 (13.0)
3	823 (17.2)	267 (16.5)	1090 (17.0)
4	872 (18.2)	350 (21.6)	1222 (19.1)
5	1488 (31.1)	483 (29.8)	1971 (30.8)
Missing	370 (7.7)	128 (7.9)	498 (7.8)
ICE-Income quintile			
1	1542 (32.2)	483 (29.8)	2025 (31.6)
2	952 (19.9)	360 (22.2)	1312 (20.5)
3	747 (15.6)	303 (18.7)	1050 (16.4)
4	774 (16.2)	209 (12.9)	983 (15.3)
5	608 (12.7)	199 (12.3)	807 (12.6)
Missing	165 (3.4)	67 (4.1)	232 (3.6)
ICE-Race-Black quintile			
1	553 (11.5)	196 (12.1)	749 (11.7)
2	823 (17.2)	320 (19.7)	1143 (17.8)
3	997 (20.8)	294 (18.1)	1291 (20.1)
4	1184 (24.7)	414 (25.5)	1598 (24.9)
5	1067 (22.3)	330 (20.4)	1397 (21.8)
Missing	164 (3.4)	67 (4.1)	231 (3.6)
ICE-Race-Hispanic quintile			
1	559 (11.7)	196 (12.1)	755 (11.8)
2	823 (17.2)	321 (19.8)	1144 (17.8)
3	996 (20.8)	312 (19.2)	1308 (20.4)
4	1142 (23.9)	376 (23.2)	1518 (23.6)
5	1104 (23.1)	349 (21.5)	1453 (22.8)
Missing	164 (3.4)	67 (4.1)	231 (3.6)
ICE-Race-Income-Black quintile			
1	1426 (29.8)	450 (27.8)	1876 (29.3)
2	1079 (22.5)	406 (25.0)	1485 (23.2)
3	683 (14.3)	241 (14.9)	924 (14.4)
4	617 (12.9)	202 (12.5)	819 (12.8)
5	818 (17.1)	255 (15.7)	1073 (16.7)
Missing	165 (3.4)	67 (4.1)	232 (3.6)
ICE-Race-Income-Hispanic quintile			
1	1438 (30.0)	454 (28.0)	1892 (29.5)
2	1103 (23.0)	411 (25.4)	1514 (23.6)
3	710 (14.8)	258 (15.9)	968 (15.1)
4	575 (12.0)	184 (11.4)	759 (11.8)
5	797 (16.6)	247 (15.2)	1044 (16.3)
Missing	165 (3.4)	67 (4.1)	232 (3.6)
Cancer stage			
I	2014 (42.1)	917 (56.6)	2931 (45.7)
II	1000 (20.9)	262 (16.2)	1262 (19.7)
III	630 (13.2)	193 (11.9)	823 (12.8)
IV	1111 (23.2)	229 (14.1)	1340 (20.9)
Missing	33 (0.7)	20 (1.2)	53 (0.8)
Imaging			
No. of MRIs ordered, mean (SD)	0.12 (0.36)	0.18 (0.43)	0.13 (0.38)
No. of CTs ordered, mean (SD)	0.37 (0.71)	0.63 (0.96)	0.44 (0.79)

Abbreviations: ALT, alanine aminotransferase; AST, aspartate aminotransferase; CT, computed tomography; ICE, Index of Concentration at the Extremes; MRI, magnetic resonance imaging; WBC, white blood cell.

SI conversion factors: To convert creatinine to μmol/L, multiply by 88.4; bilirubin to μmol/L: 17.104; albumin to g/L: 10.0; ALT and AST to μkat/L: 0.0167; WBCs to cells × 10^9^/L: 0.001; hemoglobin to g/L: 10.0; platelets to cell × 10^9^/L: 1.0.

^a^ Race and ethnicity categories were derived from the patient electronic health records. “Other” includes those listed as such in the electronic health record along with American Indian/Alaska Native.

### Model Outcome

The outcome for the model was a binary variable indicating whether time from diagnosis to the initiation of the first cancer-directed therapy (surgery, radiation therapy, or systemic therapy) was greater than 60 days. This interval was chosen based on existing quality metrics^12,14^ and clinical outcomes data.^2^ Specifically, previous studies that have analyzed time to first treatment as a continuous variable identified an association between delays in 60-day intervals and increases in cancer-specific mortality.^2^ The 60-day threshold for treatment initiation has also been incorporated into Commission on Cancer quality measures.^14^

### Statistical Analysis

#### Main Analysis

We developed machine learning models to estimate a treatment delay of greater than 60 days. Machine learning models have several potential advantages. Among those, they can facilitate variable selection in the setting of high numbers of input variables. To account for overfitting, data were split such that 80% of the data was used for training and 20% was used for testing. All models were developed exclusively using the training data set and then performance was separately evaluated in the test data set. We evaluated and compared 4 different model types: group least absolute shrinkage and selection operator (LASSO),^22^ bayesian additive regression tree,^23^ gradient boosting,^24^ and random forest.^25^ Further details on model fitting are provided in eMethods in Supplement 1.

Our primary performance metric was area under the receiver operating characteristic curve (AUC-ROC). The AUC-ROC was selected because it is a threshold independent measure of performance^26^ and is familiar to clinicians.^27^ We also evaluated several other performance metrics across a range of estimated probability cutoffs for classifying individuals as positive or negative, including sensitivity, specificity, positive predictive value (PPV), and alert rate (the proportion of the population that is classified as positive by the model). A range of cutoffs was used because, in contrast to AUC-ROC, these other metrics are threshold dependent (meaning the value of these metrics depends on the estimated probability cutoff value used to classify individuals as positive or negative). Model calibration was assessed by plotting the observed vs estimated probabilities for 10 equally sized bins, and Cox intercepts and slopes were also assessed.^10^ Neighborhood variables, laboratory variables, and stage were missing for some patients (Table 1). Missing data were handled as described in eMethods in Supplement 1.

After initial model development, we used 3 factors to select the optimal model for subsequent analyses: (1) discrimination, (2) calibration, and (3) interpretability/simplicity. The group LASSO model was selected as our final model due to its comparatively high discrimination (eTable in Supplement 1), superior calibration (eFigure 2 in Supplement 1), because it was the most interpretable due to clinician familiarity with regression-based models, and because zero imputation can be done easily in real time (eMethods in Supplement 1).

Following development of the final model, we evaluated it for equity by testing for unequal performance suggestive of algorithmic bias.^28^ We evaluated model performance for the following populations: Black patients, patients with race and ethnicity other than non-Hispanic White, White/non-Hispanic patients, and those who live in the most disadvantaged neighborhoods by quintile of the Yost Index. For these analyses, the test data set was limited to patients with each of these characteristics. Additional categories of underserved populations (eg, other categories of race, Hispanic patients only) were not evaluated due to smaller numbers in the test data set and concerns about model stability.

To determine the contribution of SDOH variables, we fit models with and without neighborhood-level SDOH variables. To allow for the possibility that these variables may hold differing degrees of information for different sociodemographic groups, we fit models with and without SDOH variables both in the overall population and also separately in each of the subgroups to assess biases, as noted above.

#### Sensitivity Analyses

As different care processes and factors may influence timely treatment for patients diagnosed within vs external to the treating institution, sensitivity analyses were performed in which the sample was limited to each of these groups.

We followed the Transparent Reporting of a Multivariable Prediction Model for Individual Prognosis or Diagnosis (TRIPOD) reporting guideline.^29^ All analyses were performed using R, version 4.2.1 (R Foundation for Statistical Computing), and Python, version 3.9 (Python Software Foundation).

## Results

### Study Population Characteristics

A total of 6409 patients (mean [SD] age, 62.8 [12.5] years; 4321 [67.4%] female) were included in the study population. The most common cancer types were breast (2576 [40.2%]), lung (1738 [27.1%]) and kidney (1059 [16.5%]). Among the 6409 patients, 229 (3.6%) were Asian or Pacific Islander; 850 (13.3%) were Black; 4975 (77.6%) were White; 124 (1.9%) were other race; and 231 (3.6%) were of unknown race. English was the preferred language for 5830 (90.1%), and 794 (12.4%) came from the most economically disadvantaged quintile based on the Yost Index (Table 1). The index cancer was the first malignant neoplasm for 5145 (80.3%), and 4944 (77.1%) patients were diagnosed outside of the treating hospital. A total of 1621 patients (25.3%) experienced a diagnosis to treatment interval of greater than 60 days.

### Model Performance

The AUC-ROC (95% CI) for the group LASSO model was 0.713 (0.679-0.745). Regression coefficients (reflecting a variable’s relative importance in estimating the likelihood of delay) are presented in Table 2 for the variables that were selected by the model (those not listed were not selected). Patients were less likely to experience a delay if they were diagnosed at the treating institution; the index cancer was their first malignant neoplasm; they were Asian or Pacific Islander or White (as compared with Black); privately insured (vs Medicaid or uninsured); or had later-stage disease. Patients were more likely to experience a delay if they had comorbidities (eg, heart failure, vascular disease, pulmonary disease) or an elevated creatinine level. Compared with the least disadvantaged groups, patients were more likely to experience a delay if they came from the most disadvantaged extremes of neighborhood deprivation, privilege (Index of Concentration at the Extremes [ICE]-Income), as well as neighborhoods with a high concentration of Hispanic residents (ICE-Race-H). This pattern was not observed for neighborhood concentration of Black residents (ICE-Race-B), where the quintile with the highest concentration of Black residents was associated with less of a delay in treatment. Patterns were less consistent for mid-level quantiles.

**Table 2.** Group LASSO Model Regression Coefficients

Variable^a^	Log odds ratio^b^
Intercept	−0.986
Primary site	
Colon	−0.507
Kidney	0.430
Lung	0.274
Rectum	0.165
Bladder	−0.426
Breast	0 [Reference]
Cancer diagnosed at treating institution	−1.165
First malignant neoplasm	−0.356
Race^c^	
Asian/Pacific Islander	−0.316
Black	0.429
White	0 [Reference]
Other	0.103
Unknown	−0.498
Primary language	
Not English	0.009
Unknown	0.006
English	0 [Reference]
Insurance	
Medicaid	0.424
Medicare	0.176
None documented	0.445
Other	0.178
Private	0 [Reference]
Yost Index quintile	
1 (Most disadvantaged)	0.038
2	0.017
3	−0.022
4	0.046
5	0 [Reference]
Missing	0.012
ICE-Income quintile	
1	0 [Reference]
2	0.091
3	0.184
4	−0.100
5 (Most disadvantaged)	0.076
Missing	0.073
ICE-Race-Black quintile	
1	0 [Reference]
2	0.130
3	−0.085
4	0.020
5 (Most disadvantaged)	−0.063
Missing	0.027
ICE-Race-Hispanic quintile	
1	0 [Reference]
2	0.047
3	0.0003
4	−0.017
5 (Most disadvantaged)	0.0003
Missing	0.013
Stage	
I	0 [Reference]
II	−0.442
III	−0.494
IV	−0.757
Missing	0.231
Age	0.007
Comorbidities	
Congestive heart failure	0.624
Peripheral vascular disorder	0.444
Hypertension	0.209
Paralysis	1.110
Chronic pulmonary disease	0.325
Diabetes	0.010
Kidney failure	0.085
Peptic ulcer disease	0.589
AIDS/HIV	0.006
Rheumatoid arthritis/collagen vascular disease	0.011
Obesity	0.089
Fluid and electrolyte disorder	0.034
Deficiency anemia	−0.105
Drug abuse	0.153
Laboratory values	
Creatinine	0.079
Creatinine missing	0.036
Albumin	−0.007
Albumin missing	−0.240
ALT	−0.0003
ALT missing	−0.022
WBCs (per thousand)	−0.030
WBCs missing	−0.349
Platelets	−0.001
Platelets missing	−0.216
Percent transportation	−0.002
Percent transportation missing	−0.003
MRI count	0.015
CT count	0.360

Abbreviations: ALT, alanine aminotransferase; CT, computed tomography; ICE, Index of Concentration at the Extremes; LASSO, least absolute shrinkage and selection operator; MRI, magnetic resonance imaging; WBC, white blood cell.

^a^ Variables not listed in this table were not selected.

^b^ More positive log odds ratios indicate a greater likelihood of delay, while more negative log odds ratios indicate a lower likelihood of delay. Variables closer to zero had less of an influence on model estimations of risk.

^c^ Race and ethnicity categories were derived from the patient electronic health records. “Other” includes those listed as such in the electronic health record along with American Indian/Alaska Native.

### Model Performance in Vulnerable Subgroups

As compared with the overall population, AUC-ROC was lower in Black patients, patients with race and ethnicity other than non-Hispanic White, and those living in the most disadvantaged neighborhoods (by quintile of the Yost Index) (Table 3). However, confidence intervals were wide.

**Table 3.** Group LASSO Model Performance by Sociodemographic Subgroups (Complete Model)

Population	AUC-ROC (95% CI)
Overall population	0.713 (0.679-0.745)
Black participants	0.641 (0.554-0.723)
Participants with race and ethnicity other than non-Hispanic White	0.664 (0.595-0.729)
White non-Hispanic participants	0.732 (0.691-0.769)
Most disadvantaged quintile of Yost Index	0.665 (0.563-0.757)

Abbreviations: AUC-ROC, area under the receiver operating characteristic curve; LASSO, least absolute shrinkage and selection operator.

### Contribution of Neighborhood-Level SDOH Variables

Though the group LASSO model selected neighborhood-level SDOH variables as contributing variables, AUC-ROC was similar when models were fit with these variables and when they were excluded. This was true both in the overall study population and across the sociodemographic subgroup populations analyzed (Table 4).

**Table 4.** Group LASSO Model Performance When Fit With and Without Neighborhood Social Determinants of Health (SDOH) Variables, by Population

Population	AUC-ROC (95% CI)	
	With SDOH	Without SDOH
Total study population	0.713 (0.679-0.745)	0.714 (0.682-0.747)
Black participants	0.640 (0.554-0.727)	0.647 (0.560-0.733)
Participants with race and ethnicity other than non-Hispanic White	0.641 (0.573-0.712)	0.643 (0.573-0.715)
White non-Hispanic participants	0.727 (0.686-0.764)	0.727 (0.688-0.766)
Most disadvantaged quintile of Yost Index	0.637 (0.538-0.725)	0.649 (0.550-0.741)

Abbreviations: AUC-ROC, area under the receiver operating characteristic curve; LASSO, least absolute shrinkage and selection operator.

### Additional Accuracy Metrics and Trade-Offs by Cutoff Value for Positivity

Table 5 presents the sensitivity, specificity, PPV, and alert rate across a range of cutoff values at which to consider a patient at risk of delay for the group LASSO model. We selected these cutoff values based on the distribution of estimated probabilities, aiming to balance the 4 metrics. Specificity was high across cutoff values, while sensitivity exhibited greater variation by cutoff value. Decreasing the cutoff value from 0.5 to 0.4 increased sensitivity by 86%, while only reducing specificity from 0.954 to 0.902 and PPV from 0.548 to 0.514. However, the alert rate doubled with this change in threshold.

**Table 5.** Group LASSO Model Accuracy Metrics by Cutoff

Estimated probability cutoff	Sensitivity (95% CI)	Specificity (95% CI)	Positive predictive value (95% CI)	Alert rate (95% CI)
0.3	0.521 (0.468-0.575)	0.788 (0.761-0.814)	0.457 (0.406-0.511)	0.290 (0.267-0.315)
0.4	0.304 (0.255-0.359)	0.902 (0.833-0.920)	0.514 (0.448-0.588)	0.151 (0.132-0.171)
0.5	0.163 (0.122-0.206)	0.954 (0.940-0.966)	0.548 (0.448-0.647)	0.076 (0.061-0.091)
0.6	0.077 (0.049-0.107)	0.985 (0.976-0.922)	0.632 (0.481-0.781)	0.031 (0.022-0.041)

Abbreviation: LASSO, least absolute shrinkage and selection operator.

### Sensitivity Analyses

Model performance was similar in patients diagnosed internally vs externally: AUC-ROC (95% CI) of 0.715 (0.622-0.803) vs 0.701 (0.666-0.735), respectively.

## Discussion

Machine learning models incorporating clinical, demographic, and neighborhood-level SDOH data effectively identified patients who were likely to experience a delay in initiating their cancer treatment. While SDOH variables were selected by models as contributing variables, discrimination was similar in models fit with and without these variables. When examining performance across population subgroups, analyses suggested that estimations may be less reliable in certain vulnerable groups than in the overall population. Taken together, our findings suggest that machine learning models incorporating clinical and SDOH information are feasible and can identify patients at risk of treatment delay. However, the optimal and most equitable manner in which to incorporate SDOH variables into such models requires further investigation.

Despite the increasing prevalence of treatment delays in many cancers,^1,10^ to our knowledge, our study is the first machine learning model to apply a predictive rather than an explanatory framework^19^ to this issue. Previous work has shown that machine learning models incorporating clinical variables can be successful in evaluating the probability of an outcome and can supplement existing oncology clinical workflows and care.^30,31^ Our findings suggest that machine learning models can similarly be used to identify patients at risk of treatment delays and builds on our prior work in socioeconomically disadvantaged groups.^32^

Our model selected neighborhood-level SDOH variables, which are not routinely available in the EHR as variables. However, their contribution to model performance is limited. This is evidenced both by the lower odds ratio estimates for individual SDOH variables and by similar AUC-ROC values when fit without these variables. One possible reason for this is that SDOH variables may add limited value on top of other variables already included in the model (eg, race). Race and economic variables are frequently correlated, and median income (but not race) was associated with delays in treatment in other studies with a higher proportion of patients from underserved race and ethnicity groups.^11^ While our mostly White, middle class, and insured study sample (all of which are associated with a lack of treatment delay^1,7,8^) may have limited our ability to evaluate the value of SDOH variables in estimating the probability of a delay, it is notable that inclusion of SDOH variables neither improved model performance in the total study population nor in sociodemographic subgroups.

Another potential explanation is that even though SDOH indices are associated with health outcomes,^33,34^ the complexity of the neighborhood-outcome relationship^35^ makes them less robust in the current context. Prior literature has suggested that a complex relationship exists between deprivation indices and outcomes. Studies have found that outcomes are worse in the most deprived areas, but that differences between middle quantiles for measures of deprivation are less pronounced.^36^ In our study, the lowest likelihood of delay came in one of the middle quintiles (Q3 or Q4) of the neighborhood variables. While not directly evaluated here, it is likely that a number of factors contributed to this. Higher likelihood of delay at the lower extremes of income may be driven by chronic stress caused by financial insecurity for those residing in low-income, high-deprivation areas. Meanwhile, advantaged patients may have the resources to obtain additional opinions and transfer care.^37^ Transfers can delay treatment, and in our study a diagnosis outside of the treating institution indicated a higher probability of delay. It is also possible that, in both cases (financial security or insecurity), delays could be driven by an inability to seek health care due to demands of employment (long hours regardless of pay).^38^ One potential area for future research is whether individually weak SDOH variables might be combined into a stronger composite score that has a stronger association with probability of delay, similar to polygenic risk scores or combined classification algorithms involving serum biomarkers.^39^

Our finding that model performance was potentially less reliable in vulnerable subgroups, despite the addition of SDOH variables, adds to the growing literature that careful attention to unintended consequences is needed when attempting to estimate risk. Work in fields outside of oncology has shown that preexisting disparities may affect model perceptions of risk and lead to inequitable resource allocation^40^ or therapeutic intensity.^41^ Mindful that these issues could also affect risk estimations regarding receipt of cancer treatment, we specifically examined model performance in several vulnerable subgroups. We found that point estimates of model performance were consistently lower as compared with the general population, although confidence intervals were wide. Poorer performance in vulnerable subgroups at high risk for treatment delays^1,7,8^ has implications regarding how the use of such a model might influence equity, as these groups also derive benefit from bringing additional resources to bear.^42,43^ Future investigations should be considered in which the training data set contains a higher proportion of vulnerable sociodemographic groups to determine whether greater subsample sizes can improve accuracy across populations.

### Limitations

Our study has limitations. First, variables not available in EHR-derived data sets may affect access to and timing of cancer therapy. To mitigate this issue, we incorporated neighborhood-level variables, which capture proxy information about socioeconomic status that is not routinely available in the EHR. However, these are not a comprehensive picture of an individual’s barriers to treatment. Variables potentially affecting treatment, such as the timeliness of appropriate molecular testing,^44^ were not able to be evaluated as part of the present study. Other sources of data, study populations, and variables not included here can be incorporated into future investigations to determine whether model performance can be improved. There are also potentially other unmeasured psychosocial and logistical factors, which were not captured in our data set. However, it is notable that despite internal vs external diagnosis being strongly associated with probability of delay, and the potentially heterogeneous barriers faced by the 2 groups, our model performed similarly for both populations. Third, the time between diagnosis and the start of cancer treatment does not include the time prior to a patient’s presentation for symptoms or screening. However, the diagnostic to treatment interval is a frequently used and clinically important measure for several reasons: (1) it is correlated with clinical outcomes and is the basis for quality measures,^1,2,14^ (2) it has the potential to be directly influenced by a patient’s cancer care team, (3) it can be captured in EHR and registry data, and (4) other efforts are under way to enhance routine cancer screening. Lastly, while we included a broad population of patients across several common solid tumors, our results are applicable to the population studied here, and factors not associated with probability of delay in the current context may be in others.

## Conclusions

In this cohort study, findings suggest that a regression-based group LASSO model incorporating EHR and SDOH data can identify the likelihood that a patient will experience a delay initiating their cancer-directed therapy. Future work should focus on additional ways to incorporate SDOH data, which may improve model performance, particularly in vulnerable populations.

## Notes

Supplement 1.
eMethods
**eFigure 1:** Patient Flow Diagram **eFigure 2:** Calibration Plots **eTable:** Model Performance in the Total Cohort for Each Model Type
eReferences

Supplement 2.
Data Sharing Statement
