# Supplementary material for: Development of a Multilevel Model to Identify Patients at Risk for Delay in Starting Cancer Treatment
Source: JAMA Netw Open. 2023 Aug 14;6(8):e2328712. doi: 10.1001/jamanetworkopen.2023.28712 (PMC10425824; doi:10.1001/jamanetworkopen.2023.28712)
Supplement: Supplement 1. — eMethods eFigure 1: Patient Flow Diagram eFigure 2: Calibration Plots eTable: Model Performance in the Total Cohort for Each Model Type eReferences [file jamanetwopen-e2328712-s001.pdf]

Supplemental Online Content

Frosch ZAK, Hasler J, Handorf E, et al. Development of a multilevel model to identify patients at risk for delay in starting cancer treatment. *JAMA Netw Open.* 2023;6(8):e2328712. doi:10.1001/jamanetworkopen.2023.28712

eMethods.

eFigure 1. Patient Flow Diagram

eFigure 2. Calibration Plots

eTable. Model Performance in the Total Cohort for Each Model Type

eReferences

This supplemental material has been provided by the authors to give readers additional information about their work.

eMethods.

Social Determinants of Health Variables and Domains:

1) Economic stability domain:
    a. Yost deprivation index, a composite deprivation score including poverty, education, and employment.[1] Lower scores on this index indicate more economic disadvantage.
    b. Index of concentration at the extremes (Ice)-race and income variables, a simultaneous measure of economic and racial/ethnic concentration.[2] Higher scores on these variables indicate greater disadvantage and/or segregation.
    c. Median household income
    d. Percentage of households below the poverty line
    e. Percentage of the civilian labor force of 16 years of age who are employed
2) Social context domain
    a. Percentage of residents born outside the United States
3) Education domain
    a. Percentage of adults over 25 years of age with less than a high school education
4) Access domain
    a. Percentage of workers who commute by public transit
5) Built environment domain
    a. Percentage of individuals ages 5 and older who lived in the same house one year ago
    b. Percentage renter occupied housing units.

Variables Included in the Machine Learning Models

Elixhauser co-morbidities were derived from ICD codes contained in the electronic health record as previously described.[3] The first laboratory value following diagnosis (but prior to treatment initiation) was included for each of the following studies: white blood cell count (WBC, 10^3/µL), hemoglobin (g/dl), platelets (10^3/µL), creatinine (mg/dl), total bilirubin (mg/dl), albumin (g/dl), aspartate aminotransferase (AST, U/L), and alanine aminotransferase (ALT, U/L). Imaging variables included the number of distinct CT scans or MRI orders placed in the EMR prior to starting treatment.

**Variable Name**	**Variable Type**
Primary Site	Categorical
Age at Diagnosis	Continuous
Cancer Diagnosed at Treating Institution	Binary
First Malignancy	Binary
Sex	Binary
Race	Categorical
Ethnicity	Categorical
Primary Language	Categorical
Insurance	Categorical
Congestive Heart Failure Diagnosis	Binary
Cardiac Arrhythmias Diagnosis	Binary
Valvular Disease Diagnosis	Binary
Pulmonary Circulation Disorder Diagnosis	Binary
Peripheral Vascular Disorder Diagnosis	Binary

Hypertension Diagnosis	Binary
Paralysis Diagnosis	Binary
Neurological Disorder Diagnosis	Binary
Chronic Pulmonary Disease Diagnosis	Binary
Diabetes Diagnosis	Binary
Hypothyroidism Diagnosis	Binary
Renal Failure Diagnosis	Binary
Liver Disease Diagnosis	Binary
Peptic Ulcer Disease Diagnosis	Binary
AIDS/HIV Diagnosis	Binary
RA/Collagen Vascular Disease Diagnosis	Binary
Coagulopathy Diagnosis	Binary
Obesity Diagnosis	Binary
Weight Loss Diagnosis	Binary
Fluid and Electrolyte Disorder Diagnosis	Binary
Blood Loss Anemia Diagnosis	Binary
Deficiency Anemia Diagnosis	Binary
Drug Abuse Diagnosis	Binary
Psychoses Diagnosis	Binary
Depression Diagnosis	Binary
First Creatinine Value Post Diagnosis	Continuous
First Bilirubin Value Post Diagnosis	Continuous
First Albumin Value Post Diagnosis	Continuous
First AST Value Post Diagnosis	Continuous
First ALT Value Post Diagnosis	Continuous
First WBC Value Post Diagnosis	Continuous
First Hemoglobin Value Post Diagnosis	Continuous
First Platelets Value Post Diagnosis	Continuous
Household Income	Continuous
Percent Poverty	Continuous
Percent Renter Occupied Households	Continuous
Percent Born Outside US	Continuous
Percent Education Less than High School	Continuous
Percent Employed	Continuous
Percent Transportation Public Transit	Continuous
Percent Living in Same House as 1 Year Ago	Continuous
Yost Quintile	Categorical
Ice Income Quintile	Categorical
Ice Race Black Quintile	Categorical
Ice Race Hispanic Quintile	Categorical
Ice Race-Income Black Quintile	Categorical
Ice Race-Income Hispanic Quintile	Categorical
Stage	Categorical
Total MRI	Continuous
Total CT	Continuous

Model Fitting: Additional Methods by Model

Group Lasso
For regression-based models (group lasso), the optimal regularization parameter was selected using 10-fold cross-validation. Tuning on alpha (vs use of L1 penalty only) did not improve model performance

Groupings included in the group lasso model. All variables not included in this table were the only variables in their groups.

Group	Variables Included
Primary Site	Primary Site Colon, Primary Site Kidney, Primary Site Lung, Primary Site Rectum, Primary Site Bladder, Primary Site Breast (Reference)
Race	Asian, Black, White (Reference), Other, Unknown,
Ethnicity	Hispanic or Latinx, Not Hispanic or Latinx
Primary Language	Not English, Unknown, English (Reference)
Insurance	Medicaid, Medicare, None, Other, Private
Yost Quintile	Yost Q1, Yost Q2, Yost Q3, Yost Q4, Yost Q Missing Yost Q5 (Reference)
Ice Income Quintile	Ice Inc Q1 (Reference), Ice Inc Q2, Ice Inc Q3, Ice Inc Q4, Ice Inc Q5, Ice Inc Q Missing
Ice Race Black Quintile	Ice Race B Q1 (Reference), Ice Race B Q2, Ice Race B Q3, Ice Race B Q4, Ice Race B Q5, Ice Race B Q Missing
Ice Race Hispanic Quintile	Ice Race H Q1 (Reference), Ice Race H Q2, Ice Race H Q3, Ice Race H Q4, Ice Race H Q5, Ice Race H Q Missing
Ice Race-Income Black Quintile	Ice RIB Q1 (Reference), Ice RIB Q2, Ice RIB Q3, Ice RIB Q4, Ice RIB Q5, Ice RIB Q Missing
Ice Race-Income Hispanic Quintile	Ice RIH Q1 (Reference), Ice RIH Q2, Ice RIH Q3, Ice RIH Q4, Ice RIH Q5, Ice RIH Q Missing
Stage	Stage 1 (Reference), Stage 2, Stage 3, Stage 4, Stage Missing
Creatinine	First Creatinine Post Diagnosis, First Creatinine Post Diagnosis Missing
Bilirubin	First Bilirubin Post Diagnosis, First Bilirubin Post Diagnosis Missing
Albumin	First Albumin Post Diagnosis, First Albumin Post Diagnosis Missing
AST	First AST Post Diagnosis, First AST Post Diagnosis Missing
ALT	First ALT Post Diagnosis, First ALT Post Diagnosis Missing
WBC	First WBC Post Diagnosis, First WBC Post Diagnosis Missing

Group	Variables Included
Hemoglobin	First Hemoglobin Post Diagnosis, First Hemoglobin Post Diagnosis Missing
Platelets	First Platelets Post Diagnosis, First Platelets Post Diagnosis Missing
Household Income	Household Income, Household Income Missing
Percent Poverty	Percent Poverty, Percent Poverty Missing
Percent Renter Occupied Households	Percent Renter Occupied Households, Percent Renter Occupied Households Missing
Percent Born Outside US	Percent Born Outside US, Percent Born Outside US Missing
Percent Education Less than High School	Percent Education Less than High School, Percent Education Less than High School Missing
Percent Employed	Percent Employed, Percent Employed Missing
Percent Transportation Public Transit	Percent Transportation Public Transit, Percent Transportation Public Transit Missing
Percent Same House Percent Living in Same House as 1 Year Ago	Percent Living in Same House as 1 Year Ago, Percent Living in Same House as 1 Year Ago

Hyper-parameters for Gradient Boosting and Random Forest Models
Hyper-parameter tuning was conducting using a random grid search over 500 iterations with 5-fold cross validation. The scoring function used was ROC-AUC. Below is the hyper-parameter search space for the Random Forest and Gradient Boosting models along with the optimal values for each hyper-parameter for the full models.

**Model**	**Hyperparameter**	**Search Space**	**Optimal**
Gradient Boosting	Maximum depth of individual regression estimators	3, 5, 7, 10, 15, 20, 25, 30, 35, 40, 45, 50, 55, None	3
Gradient Boosting	Number of variables to consider when looking for best split	None, 'sqrt'	'sqrt'
Gradient Boosting	Minimum number of samples required to be at a leaf node	1, 2, 4	1
Gradient Boosting	Minimum number of samples required to split an internal node	2, 5, 10, 12, 15	10
Gradient Boosting	The number of boosting stages to perform	10, 120, 230, 340, 450, 560, 670, 780, 890, 1000	890
Gradient Boosting	The fraction of samples to be used for fitting the individual base learners	0.5, 0.8, 1	0.8
Gradient Boosting	Learning rate	0.01, 0.03111111, 0.05222222, 0.07333333, 0.09444444, 0.11555556, 0.13666667, 0.15777778, 0.17888889, 0.2	0.01
Random Forest	Number of trees	10, 120, 230, 350, 450, 560, 670, 780, 890, 1000	560
Random Forest	Number of variables to consider when looking for best split	None, 'sqrt'	'sqrt'
Random Forest	Minimum number of samples required to be at a leaf node	1, 2, 4	4
Random Forest	Minimum number of samples required to split an internal node	2, 5, 10, 12, 15	10
Random Forest	Maximum depth of tree	3, 5, 7, 10, 15, 20, 25, 30, 35, 40, 45, 50, 55, None	7

Hyper-Parameters for BART Model

A BART model with logistic latents was fit using the BART R package. The number of trees was set to 200. 100 MCMC samples were discarded for burn-in, and 1,000 MCMC samples were returned by the algorithm. The default values were used for the remaining parameters and the mean of the 1,000 posterior samples for the model estimated on the test set was used. For additional details, refer to the BART R package vignette.[4]

Handling of Missing Data:

Initially, missing data was handled via predictive mean matching[5] for all variable types. For group lasso, we additionally tested a simpler strategy that is easier to implement in real time: we imputed missing values of continuous variables as 0 along with a missing indicator for that variable and, for categorical variables, included a missing category. Zero imputation vs predictive mean matching did not meaningfully affect model performance (AUC [95% CI]): 0.705 [0.671-0.739] for predictive mean matching vs AUC [95% CI]): 0.713 [0.679-0.745] for zero imputation). As zero imputation may be more useful for obtaining real-time estimations of risk, those results are presented.

eFigure 1. Patient Flow Diagram

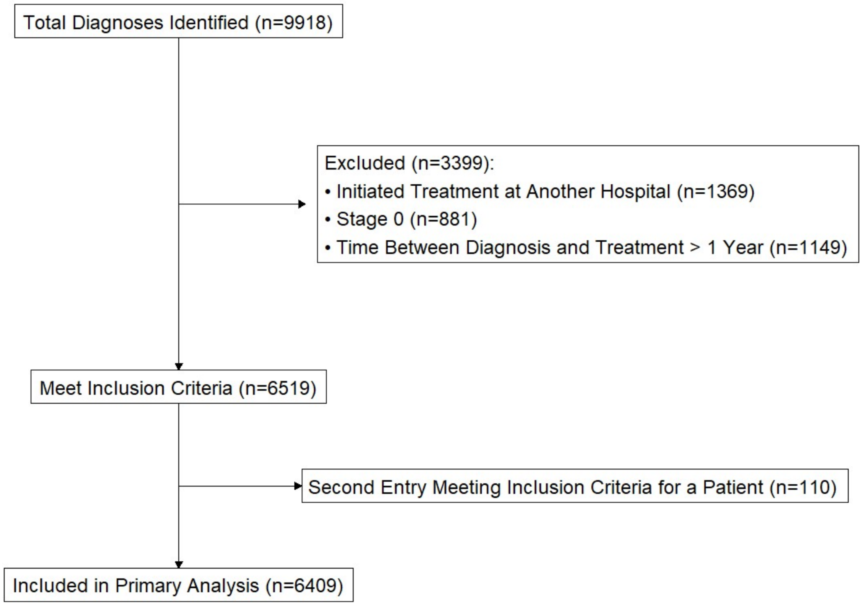

eFigure 2. Calibration Plots. The group lasso model was well calibrated. Random forest and gradient boosting models generally underestimated risk (based on calibration intercept >0) while the BART model overestimated risk (Based on calibration intercept <0).

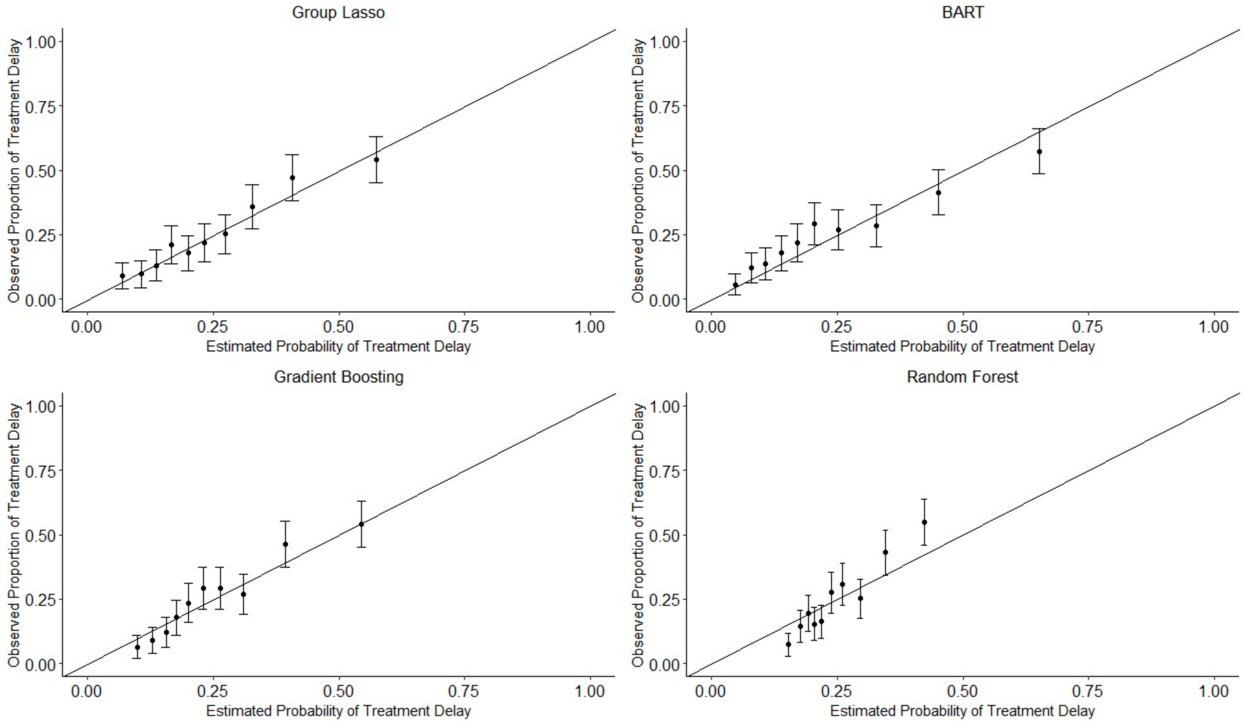

eTable. Model Performance in the Total Cohort for Each Model Type

**Model**	**AUC-ROC (95% CI)**
Group Lasso	0.713 (0.679-0.745)
BART	0.710 (0.678-0.742)
Gradient Boosting	0.715 (0.683-0.746)
Random Forest	0.691 (0.656-0.724)

eReferences

1. Yost K, Perkins C, Cohen R, Morris C, Wright W. Socioeconomic status and breast cancer incidence in California for different race/ethnic groups. *Cancer Causes Control*. Oct 2001;12(8):703-11. doi:10.1023/a:1011240019516

2. Krieger N, Waterman PD, Spasojevic J, Li W, Maduro G, Van Wye G. Public Health Monitoring of Privilege and Deprivation With the Index of Concentration at the Extremes. *Am J Public Health*. Feb 2016;106(2):256-63. doi:10.2105/AJPH.2015.302955

3. Quan H, Sundararajan V, Halfon P, et al. Coding algorithms for defining comorbidities in ICD-9-CM and ICD-10 administrative data. *Med Care*. Nov 2005;43(11):1130-9. doi:10.1097/01.mlr.0000182534.19832.83

4. Sparapani R, Spanbauer C, McCulloch R. Nonparametric Machine Learning and Efficient Computation with Bayesian Additive Regression Trees: The BART R Package. *Journal of Statistical Software*. 2021;97(1)doi:10.18637/jss.v097.i01

5. Van Buuren S, Groothuis-Oudshoorn K. mice: Multivariate imputation by chained equations in R. *Journal of Statistical Software*. 2011;45:1-67.
