# Supplementary material for: Development of a Multilevel Model to Identify Patients at Risk for Delay in Starting Cancer Treatment
Source: JAMA Netw Open. 2023 Aug 14;6(8):e2328712. doi: 10.1001/jamanetworkopen.2023.28712 (PMC10425824; doi:10.1001/jamanetworkopen.2023.28712)
Supplement: Supplement 2. — Data Sharing Statement [file jamanetwopen-e2328712-s002.pdf]

Data Sharing Statement

Frosch. Development of a Multilevel Model to Identify Patients at Risk for Delay in Starting Cancer Treatment. *JAMA Netw Open*. Published August 14, 2023.
doi:10.1001/jamanetworkopen.2023.28712

Data
Data available: No

Additional Information
Explanation for why data not available: Data definitions are included in the Supplement. Modeling code will be made available on request to the authors. Individual patient-level data will not be available due to privacy concerns and regulatory compliance.
